# Cardiovascular toxicity from therapies for light chain amyloidosis

**DOI:** 10.3389/fcvm.2023.1212983

**Published:** 2023-07-05

**Authors:** Paolo Morfino, Alberto Aimo, Vincenzo Castiglione, Michela Chianca, Giuseppe Vergaro, Carlo Maria Cipolla, Antonella Fedele, Michele Emdin, Iacopo Fabiani, Daniela Cardinale

**Affiliations:** ^1^ Interdisciplinary Center for Health Sciences, Scuola Superiore Sant’Anna, Pisa, Italy; ^2^ Cardiology Division, Fondazione Toscana Gabriele Monasterio, Pisa, Italy; ^3^ Cardioncology Unit, Cardioncology and Second Opinion Division, European Institute of Oncology, I.R.C.C.S., Milan, Italy

**Keywords:** AL amyloidosis, cardiac amyloidosis, chemotherapy, cardiovascular toxicity, treatment

## Abstract

Amyloid light-chain (AL) amyloidosis is a hematological disorder characterized by abnormal proliferation of a plasma cell clone producing monoclonal free light chains that misfold and aggregate into insoluble fibrils in various tissues. Cardiac involvement is a common feature leading to restrictive cardiomyopathy and poor prognosis. Current first-line treatments aim at achieving hematological response by targeting the plasma cell clones, and these have been adapted from multiple myeloma therapy. Patients with AL amyloidosis often exhibit multiorgan involvement, making them susceptible to cancer therapy-related cardiovascular toxicity. Managing AL amyloidosis is a complex issue that requires enhanced knowledge of the cardio-oncological implications of hematological treatments. Future research should focus on implementing and validating primary and secondary prevention strategies and understanding the biochemical basis of oncological therapy-related damage to mitigate cardiovascular toxicity.

Amyloid light-chain (AL) amyloidosis is a hematological disease caused by the proliferation of a plasma cell clone that overproduces immunoglobulin (Ig) *λ* (75%–80%) or *κ* free light chains (FLC) that are prone to misfolding and self-aggregation into amyloid fibrils (1). These fibrils accumulate in various tissues, leading to both direct tissue damage through their intrinsic proteotoxicity and architectural disruption (2).

The immunophenotype of plasma cells involved in AL amyloidosis is not much different from multiple myeloma (MM) and monoclonal gammopathy of uncertain significance (MGUS), even if the plasma cell burden, namely the bone marrow percentage of plasma cells, is generally lower than in MM (2, 3). The first-line treatment options for AL amyloidosis aim at obtaining a hematological response, defined as normalization of FLC ratio or reduction of differential FLC (difference between amyloidogenic and uninvolved circulating FLC), because the disruption of abnormal plasma cell clones results in slower disease progression and reduced amyloid deposition (4). On the other side, since the cardiac amyloid burden is the main determinant of prognosis, supportive care directed to cardiac dysfunction has a crucial role in patients with AL amyloidosis (2).

Treatment of AL amyloidosis has traditionally been readapted from that of MM. However, most MM cancer therapy regimens exhibit a potential cardiovascular (CV) toxicity leading to enhanced risk of heart failure (HF), arrhythmias and vascular disease (Figure 1) (5). Additionally, patients with AL amyloidosis often display a multiorgan involvement which makes them fragile and susceptible to cancer therapy-related CV toxicity (CTR-CT) (6).

**Figure 1:**
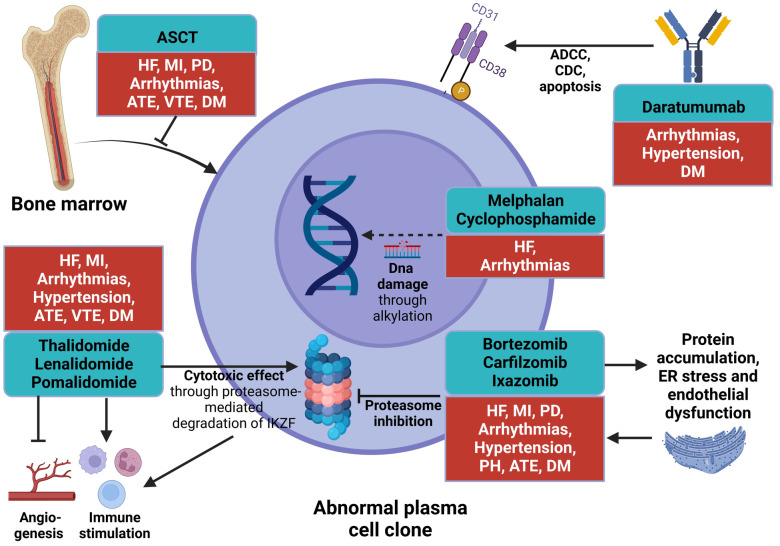
Schematic of therapeutic targets and cardiovascular toxicity in light chain amyloidosis. The Figure summarizes the targets, mechanisms of action and cardiovascular toxicities of the most frequently used drugs in AL amyloidosis. PIs impair the function of the proteasome, inducing protein accumulation. IMiDs induce the proteasome-mediated degradation of IKZF transcription factors and exert both direct and indirect (through stimulation of immune response and anti-angiogenetic effect) cytotoxic effect. Daratumumab causes ADCC, CDC, and cellular apoptosis by targeting CD38 on plasma cells. Alkylating agents induce DNA damage, thus inhibiting cellular transcription and replication. ASCT consists of autologous bone marrow substitution, which represses the proliferation of abnormal plasma cell clones. ADCC, antibody-dependent cell cytotoxicity; AL, light chain amyloidosis; ASCT, autologous stem cell transplant; ATE, arterial thromboembolism; CDC, complement-dependent cytotoxicity; DM, diabetes mellitus; HF, heart failure; IMiD, immunomodulatory drug; MI, myocardial infarction; PD, pericardial disease; PH, pulmonary hypertension; PI, proteasome inhibitor; VTE, venous thromboembolism.

This review tries to elucidate the clinical manifestations and management of CV adverse effects (AEs) caused by cancer therapy in patients with AL amyloidosis.

## AL amyloidosis

AL amyloidosis is a rare condition, with 20-year incidence and prevalence rates of approximately 10 and 51 cases per million population, but it still represents the most common form of systemic amyloidosis (3, 7). Interestingly, although AL amyloidosis is considered 5–10-fold less common than MM, about 10%–15% of patients with a diagnosis of MM will develop AL amyloidosis, and a similar proportion of AL amyloidosis patients will manifest MM (7). Patients with a MGUS exhibit a 8.8-fold higher risk of developing AL amyloidosis compared to the general population (8). AL amyloidosis also represents one of the two most common causes of cardiac amyloidosis (CA), along with transthyretin amyloidosis, together accounting for 90%–95% of all cases of CA (9). Indeed, cardiac involvement can be detected in approximately 75% of AL patients (10). AL amyloidosis is usually diagnosed at a mean age of 63 years, and is slightly more common in males (55%) (7, 11).

The diagnosis of AL-CA requires the demonstration of light-chain amyloid within tissues. Suspecting CA is crucial to start the diagnostic workup for this condition. A non-invasive multiparametric echocardiography score has been proposed to detect cardiac involvement among patients with systemic AL (AL score) or in those with hypertrophic phenotype [increased wall thickness (IWT) score]. AL score included relative wall thickness, *E*/*e*′, longitudinal strain, and TAPSE, with an optimism-corrected area under the ROC curve (AUC) of 0.905; whereas the IWT score included relative wall thickness, *E*/*e*′, longitudinal strain, TAPSE and septal longitudinal systolic apex-to-base ratio, with an AUC of 0.864 (12).

Cardiac involvement is the main determinant of prognosis in AL patients, which exhibits a 3–6 month median survival in the absence of hematological treatments, while untreated patients without cardiac involvement have a median expected survival of 13 months (13–16). The mortality rate in AL amyloidosis has dramatically decreased at all stages of the disease because of therapeutic advancements. Indeed, the analysis of patient outcomes according to the date of diagnosis (1980–2020) revealed that the 6-month mortality rate reduced from 23% to 13% over the last 40 years (7, 17, 18). To date, patients treated for AL amyloidosis display a median survival rate ranging from 0.4 to 12 years (for Mayo stages I and IIIb, respectively), which strongly depends on the disease stage at diagnosis, thus opening new perspectives on the long-term AEs of hematological treatment (19, 20).

## Hematological treatment in AL amyloidosis

Treatment for AL amyloidosis relied on melphalan and steroids until the 1990s, when the combination of melphalan and autologous hematopoietic stem cell transplantation (ASCT) was introduced, emulating MM therapy with adaptations in terms of dose and schedule. Drug agents directed to specific molecular targets became available after 2,000, including immunomodulatory drugs (IMiDs) and proteasome inhibitors (PIs) (3). Daratumumab, a novel human IgG1κ monoclonal antibody (mAb) targeting CD38 on plasma cells, has been recently introduced, while antibodies directed against amyloid fibrils are under evaluation (21).

AL amyloidosis patients are primarily evaluated to assess if they meet eligibility criteria for ASCT (approximately 20%–25%) (22). High-dose melphalan (200 mg/m^2^) is recommended before ASCT, but patients with a plasma cell burden >10% may benefit from an induction therapy with chemotherapy regimens (23). Current guidelines do not recommend a maintenance therapy after transplantation, but high-dose melphalan may be considered as consolidation treatment in those not achieving complete hematological response (24).

Daratumumab-CyBorD (cyclophosphamide, bortezomib, dexamethasone) is the preferred regimen among AL amyloidosis patients ineligible for ASCT. When daratumumab is not available, a bortezomib-based regimen is recommended, such as CyBorD or BMDex (bortezomib-melphalan-dexamethasone). CyBorD may be considered as the first-line therapy, because its administration is more feasible and indicated even in patients with moderate or severe impairment of renal function (13). At least 2 cycles after the complete hematological response (normalization of FLC ratio) are recommended, while treatment can be prolonged to 6–8 cycles in patients with at least a very good partial response (differential FLC <40 mg/L) after 3 cycles (25). The drug regimen and intensity should be based on the disease and the overall status of patients. For example, patients with Mayo cardiac stage IIIb should receive dose-modified daratumumab-CyBorD or single-agent daratumumab or alternative dose-modified CyBorD or BMDex. In patients not achieving complete or very good partial response after 2–3 cycles, therapy should be reconsidered. Patients with relapsed or refractory AL amyloidosis may be treated with first- and second-generation PIs or IMiDs at lower doses than for MM, mAb, or bendamustine (mainly in IgM related AL amyloidosis with lymphoid component in the bone marrow) (25).

## Management of cancer therapy-related cardiovascular toxicity in AL amyloidosis

### Definition and risk assessment

**Figure 2:**
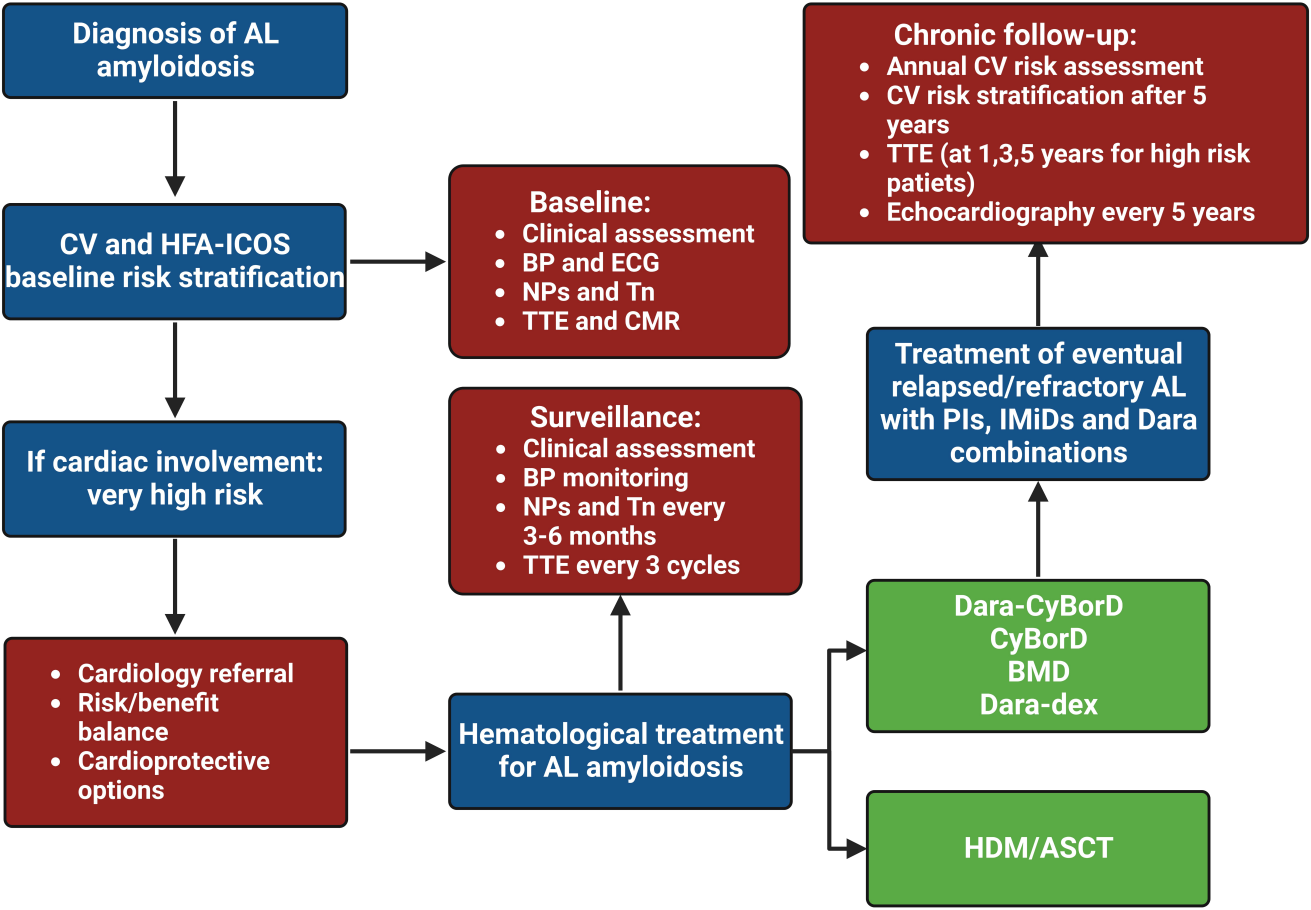
Clinical flow chart of cardio-oncological follow-up in patients with light chain amyloidosis. HDM/ASCT, high dose melphalan followed by autologous stem cell transplant; AL, light chain amyloidosis; BMD, bortezomib-melphalan-dexamethasone; BP, blood pressure; CyBorD, cyclophosphamide-bortezomib-dexamethasone; CV, cardiovascular, Dara, daratumumab; dex, dexamethasone; ECG, electrocardiogram; IMiD, immunomodulatory drug; NP, natriuretic peptide; PI, proteasome inhibitor; Tn, troponin; TTE, transthoracic echocardiogram.

The definition of CV toxicity has changed over the years. The recent evolution of cardio-oncology has led to assessing CTR-CT with more rigid criteria, as reported by the European Society of Cardiology (ESC) guidelines (Table 1) (26). The risk of CTR-CT should be established before the administration of cancer therapy (6, 27, 28). Clinical response to cancer therapy is highly variable and the risk of developing CTR-CT during or after treatment depends on several factors: age, sex, genetics, history of CV disease or cancer or cardiotoxic therapies, anomalies of cardiac biomarkers or ECG or transthoracic echocardiogram, lifestyle risk factors and their treatment (29). Only a few risk scores have been validated and the gold standard is currently represented by the Heart Failure Association of the ESC in collaboration with the International Cardio-Oncology Society (HFA-ICOS) baseline risk stratification (6, 28, 30). Patients selected for cancer therapy can be stratified into low, moderate, high and very high risk. Interestingly, patients with a diagnosis of AL and cardiac infiltration are classified as very high risk patients independently from other conditions (6). Therefore, patients with AL-CA are recommended for cardiology referral (Class I, Level C) before the administration of cancer therapy. Specialists are invited to perform a multidisciplinary discussion about the risk-benefit ratio of cancer therapy (Class I, Level C) and to evaluate potential cardioprotective strategies (Class IIa) (Figure 2) (6).

**Table 1:** Cancer therapy-related cardiovascular toxicity definitions (6, 26).

CTRCD	**Symptomatic:**
	• *Very severe*: HF requiring inotropic or mechanical circulatory support or transplantation. • *Severe*: HF hospitalization. • *Moderate*: need for intensification of diuretic and HF therapy. • *Mild*: mild HF symptoms onset without need for intensification of therapy.
	**Asymptomatic:**
	• *Severe*: LVEF reduction to <40%. • *Moderate*: LVEF reduction by ≥10 percentage points to LVEF of 40–49% OR LVEF reduction by <10 percentage points to LVEF of 40–49% AND decline in GLS by >15% from baseline OR elevation in cardiac biomarkers^a^. • Mild: LVEF ≥50% AND decline in GLS by >15% from baseline AND/OR new elevation in cardiac biomarkers^a^.
Myocarditis	• Pathohistological diagnosis: multifocal inflammatory cell infiltrates with overt cardiomyocyte loss by light microscopy of cardiac tissue samples. • Clinical diagnosis^b^: Tn elevation (new, or significant change from baseline) with 1 major criterion^c^ OR Tn elevation (new, or significant change from baseline) with 2 minor criteria^d^ after exclusion of ACS or acute infectious myocarditis.
Hypertension	• ≥140 mmHg systolic and/or ≥90 mmHg diastolic. • In patients with high CV risk: ≥130 mmHg systolic and/or ≥80 mmHg diastolic.
Arrhythmias	• QT prolongation: QTc >500 ms^e^. • For other arrhythmias: general cardiology definitions.
Vascular toxicity	• General cardiology definitions.

BNP, B-type natriuretic peptide; CMR cardiac magnetic resonance; CTRCD, cancer therapy-related cardiac dysfunction; CV, cardiovascular; HF, heart failure; LVEF, left ventricular ejection fraction; GLS, global longitudinal strain; NT-proBNP, N-terminal pro B-type natriuretic peptide; QTc, corrected QT; Tn, troponin; WMA, wall motion abnormality.

^a^ TnI/TnT >99th percentile, BNP ≥35 pg/ml, NT-proBNP ≥125 pg/ml or new significant elevation from baseline.

^b^ Clinical diagnoses should be confirmed with CMR or endomyocardial biopsy.

^c^ Major criterion: CMR diagnostic for acute myocarditis (modified Lake Louise criteria).

^d^ Minor criteria: (1) clinical syndrome (fatigue, muscle weakness, myalgias, chest pain, diplopia, ptosis, shortness of breath, orthopnea, lower extremity edema, palpitations, lightheadedness/dizziness, syncope, cardiogenic shock); (2) ventricular arrhythmia and/or new conduction system disease; (3) decline in systolic function, with or without regional WMA in a non-Takotsubo pattern; (4) other immune-related adverse events (myositis, myopathy, myasthenia gravis); suggestive CMR (meeting some but not all of the modified Lake Louise criteria).

^e^ Fridericia correction is recommended (QTcF = QT/3RR).

Collecting baseline ECG, plasma cardiac biomarkers, imaging tests, medical history and clinical examination can furtherly help physicians to delineate the personalized CTR-CT risk profile of patients (Class I, Level C). Indeed, primary and secondary prevention (management of pre-existing CV disease) strategies should be pursued to reduce the risk of future CV disease (28, 31, 32).

Evidence from several studies suggests that a healthy lifestyle, such as not smoking, maintaining physical activity, a healthy diet (high intake of vegetables, fruits and whole grains), moderate consumption of alcohol, and modest sleep duration, results in a decreased risk of transition from cancer to subsequent CV disease (33). Moreover, adult patients at high and very high risk selected for cancer therapy, such as those with AL amyloidosis, may be treated with cardioprotective drugs including statins (Class IIa, Level B) and angiotensin-converting enzyme inhibitors or angiotensin II receptor blockers plus beta-blockers (Class IIa, Level C) for primary CV prevention (6, 34). Finally, among patients with AL amyloidosis, an echocardiogram is recommended before starting cancer therapy (Class I, Level C) to assess left and right ventricular function, dilation, hypertrophy, wall motion abnormalities and other parameters which may influence the therapeutic strategy (6, 35).

### Management and chronic follow-up

CV monitoring for patients with AL-CA during hematological treatment consists of clinical assessment, with blood pressure monitoring, natriuretic peptides (NPs) and troponins (Tn) measurement every 3–6 months (Class I) (6). The routine measurement of cardiac biomarkers is crucial not only to detect CV toxicities but also to monitor the cardiac response (defined as reduction of N-terminal pro-B-type natriuretic peptide [NT-proBNP] values >30% and >300 ng/L if baseline levels >650 ng/L or New York Heart Association [NYHA] class response ≥2 if baseline class 3 or 4) to chemotherapy (36). Indeed, a BNP reduction to ≤200 ng/L during therapy is a significant predictor of improved survival in patients with AL-CA, while a >30% or >300 ng/L elevation in NT-proBNP or >33% increase in Tn reflect the progression of cardiac impairment (36, 37). Since cardiac involvement is the main predictor of mortality among AL patients, staging systems focus on parameters of heart damage. Cardiac biomarkers were also included in the most recent Mayo 2012 staging (I–IV) scoring system (TnT ≥0.025 ng/ml or high-sensitivity TnT ≥40 ng/L, NT-proBNP ≥1,800 pg/ml, and differential FLC ≥18 mg/dl) to characterize prognosis of patients with AL-CA and to properly administer risk-adapted therapies (38).

AL amyloidosis patients should also perform individual blood pressure home monitoring and an echocardiogram at the end of every 3 cycles (or every 3–6 months in patients treated with PIs) (Class IIa) (39). Finally, a specialist CV assessment is recommended for patients who exhibit new CV toxicity during and after cancer treatment (Class I, Level C) (6).

Physicians should periodically evaluate the CV toxicity risk profile of cancer survivors at the end of therapy (and 5 years after treatment in asymptomatic patients), according to recent ESC guidelines, to properly establish a follow-up plan (Class I, Level C). As reported in Table 2, stratification ranges from low to very high and divides early (<5 years) and late (>30 years) risks from completing cancer therapy (6). Asymptomatic patients should undergo an annual CV risk assessment, by collecting ECG, NPs and clinical history (Class I, Level B), while echocardiography should be performed every 5 years (or at 1, 3 and 5 years for patients at high or very high risk) (Class IIa/IIb, Level C) (6, 40). Finally, patients treated with chemo- or immunotherapy regimens may develop various CV diseases (HF, arrhythmias, pericardial disease, valvular heart disease, acute coronary syndrome, vascular disease and metabolic syndrome) which need to be treated according to relative guidelines (6).

**Table 2:** Risk stratification for adults with light chain amyloidosis after cancer therapy (6).

Risk degree	Clinical characteristics
Very high risk	Very high baseline CV toxicity risk before treatment
Early high risk	• High baseline CV toxicity risk
	• Symptomatic or asymptomatic moderate-severe CTRCD during treatment
	• High-risk ASCT^a^
Late high risk	Poorly controlled CV risk factors
Moderate risk	Moderate baseline CV toxicity risk
Low risk	• Low baseline CV toxicity risk and normal end-of-therapy cardiac assessment
	• Mild CTRCD during therapy but recovered by the end of cancer therapy

ASCT, autologous stem cell transplant; CTRCD, cancer therapy-related cardiac dysfunction; CV, cardiovascular.

^a^ High risk ASCT: pre-existing CV disease or risk factors; cancer treatment history; conditioning schemes (alkylating agents).

Cardioprotective therapy consisting of an angiotensin-converting enzyme inhibitor/angiotensin II receptor blocker and a beta-blocker, preferably carvedilol, is recommended (Class IIa) for secondary prevention of both symptomatic and asymptomatic cancer patients developing cardiac dysfunction [defined as a reduction of left ventricular ejection fraction (LVEF) ≥ 10%] according to guidelines for HF (6, 41, 42). Hypertension is another common complication of oncological treatment and, if poorly controlled despite the previously mentioned drugs, it can be treated with amlodipine or aldosterone inhibitors, avoiding negative inotropic agents (42).

In particular, patients with AL-CA amyloidosis require a supportive treatment to contrast the effects of amyloid infiltration in addition to the possible CTR-CT (4). For instance, the treatment of cardiac amyloidosis is mainly based on the management of symptoms related to HF, with loop diuretics (particularly torsemide and bumetanide), possibly coupled with mineralocorticoid receptor antagonists, being the cornerstone of therapy (41). Neuro-hormonal antagonists may be poorly tolerated in patients with AL-CA due to hypotension, conduction disorders or kidney disease (43). The management of arrhythmias and conduction disorders is complex as well. Patients with AL-CA frequently display atrial fibrillation, but catheter ablation is arduous due to the extensive atrial infiltration, with high rate of recurrence. Amiodarone and dofetilide are the preferred antiarrhythmic agents, because they do not exert negative inotropic effect (4). Moreover, anticoagulation must be prescribed to patients with atrial fibrillation regardless of the CHA2DS2-VASc score (44, 45). Although there is no evidence to support anticoagulation in patients with AL amyloidosis and sinus rhythm, patients with hematological malignancies show increased risk of thromboembolic events (45). For this reason, the use of aspirin or low molecular weight heparin is recommended in patients with MM receiving IMiDs or in those at high risk of thromboembolism (6, 46).

## Cardiovascular toxicities of therapies for AL amyloidosis

Only a limited number of studies have investigated the toxic effects of cancer therapy in AL amyloidosis. Therefore, most of the findings are based on studies conducted on patients with MM. Furthermore, the trials have used different definitions of CV toxicity, and patients with severe pre-existing comorbidities or CV disease were frequently excluded (47). In the case of AL amyloidosis, CTR-CT primarily includes cardiac dysfunction, arrhythmias, arterial vascular disease, venous thrombo-embolism and systemic hypertension, as summarized in Table 3 (48).

**Table 3:** Clinical manifestations of cancer therapy-related cardiovascular toxicity in light chain amyloidosis (49, 50).

Drugs^a^	HF	Arrhythmias	MI	ATE	VTE	Hypertension	PH	DM	PD
Bone marrow transplantation									
ASCT	C	C	U	U	U			U	U
Alkylating agents									
Melphalan	C	C							
Cyclophosphamide	U	C							
Proteasome inhibitors									
Bortezomib	U	U	U		U	U	C	U	
Carfilzomib	C	C	U		C	C	C	C	
Ixazomib	C	C						C	C
Immunomodulatory drugs									
Thalidomide	C	U	U	U	C				
Lenalidomide	C	C	C	U	C	C		C	
Pomalidomide		C	C	C	C	C		C	
Monoclonal antibodies									
Daratumumab		C				C		C	
Isatuximab	C	C				C			
Elotuzumab					C	C		C	

ASCT, autologous stem cell transplant; ATE, arterial thromboembolism; DM, diabetes mellitus; HF, heart failure; MI, myocardial infarction; PD, pericardial disease; PH, pulmonary hypertension; VTE, venous thromboembolism.

^a^ C, common (>1%); U, uncommon (<1%).

### Autologous stem cell transplantation

The increased awareness of ASCT-related toxicity as well as the growing population of transplanted survivors are an important source of concern. It is estimated that approximately two-thirds of ASCT survivors develop at least one long-term toxic effect (51), and transplanted patients after 5-year survival have a 4–9-fold higher mortality rate than the general population for 30 years (52). CV adverse events following ASCT are not common (<10%), but significantly impact on survival and quality of life (53).

Short-term CV adverse events (i.e., within 100 days) are relatively rare (0.9%–8.9%) and mainly include arrhythmias (2%–10%), congestive HF (0.4%–2.2%), and rarely myocardial infarction and pericardial effusion (54–56). Long-term toxicities consist of development of ischemic heart disease, HF, diabetes mellitus, stroke, hypertension and vascular disease (57). An observational study which involved 1,244 patients undergoing autologous transplant for a hematologic malignancy reported that the cumulative incidence of congestive HF was 4.8% after 5 years and raised to 9.1% after 15 years from ASCT (58).

The only trial comparing ASCT with a chemotherapy regimen (melphalan-dexamethasone) did not show a significant prognostic benefit from ASCT. Indeed, this small phase III study (*n* = 100) reported an ASCT-related survival rate at 3 years of 58% compared to 80% in the group treated only with chemotherapy (*p* = 0.13) (59). In a retrospective analysis comparing AL amyloidosis patients (*n* = 241) treated with ASCT vs. Mayo stage-matched non-transplanted controls, 49% of transplanted patients showed any-grade cardiac arrhythmia (60). Moreover, a study demonstrated that AL patients (*n* = 101) undergoing peripheral blood hematopoietic stem cell mobilization before ASCT had a significant risk of developing at least one AE, such as cardiac (atrial or ventricular arrhythmias, ischemic heart disease, acute HF, and hypertensive emergencies: 7%) and thromboembolic events (deep venous thrombosis and pulmonary embolism: 5%), probably due to previous growth factors administration-related release of proinflammatory cytokines (61).

### Alkylating agents

Alkylating agents inhibit the transcription and replication of DNA through alkylation, which impairs cell replication (3). Although alkylating agents are non-selective drugs associated with various AEs (mainly cytopenia and gastrointestinal toxicities), their administration combined with corticosteroids is a safe and tolerable option, but considered suboptimal (25).

#### Melphalan

Melphalan is not directly associated with CTR-CT in the context of AL amyloidosis, but treatment with ASCT and high-dose melphalan may cause atrial fibrillation and supraventricular arrhythmia, as suggested by clinical studies including patients with MM (62, 63). Among patients not eligible for transplantation, treatment with melphalan-dexamethasone (MelDex) results in significant hematological response but it is more effective when combined with bortezomib (BMDex) (64). An observational study comparing MelDex and BMDex in newly diagnosed AL patients (*n* = 109) demonstrated that treatment with melphalan is safe and well tolerated, but the addition of bortezomib was associated with a 2-fold increase in severe AEs (64).

#### Cyclophosphamide

Cyclophosphamide is a prodrug which is converted into active metabolites by cytochrome P450-2B6. Metabolites are likely to be involved in CTR-CT through various mechanisms, involving oxidative and nitrative stress, calcium imbalance, and formation of protein adducts leading to inflammation, apoptosis and dysregulation of signalling pathways (e.g., NFκB/p53/p38 MAPKs) (65). Cyclophosphamide is commonly used in MM ahead of bone marrow transplantation, but its significant CV toxicity is a contraindication for patients with AL amyloidosis (61). Indeed, patients undergoing cyclophosphamide show an increased risk of developing HF (7%–33% with a dose >150 mg/kg) (66, 67). Metabolites of cyclophosphamide may cause endothelial damage, edema, haemorrhage and thrombosis, thus resulting in myo(peri)carditis, pericardial effusion, pulmonary hypertension and acute coronary syndrome (6). In patients with advanced age and previous radiation or cardiotoxic treatment, high-dose cyclophosphamide correlates with an enhanced risk of CTR-CT (9). However, in the setting of AL amyloidosis, low-dose cyclophosphamide (500 mg *per os*) in CyBorD exhibits a good safety profile (68).

### Proteasome inhibitors

PIs tremendously improved the outcomes of patients with either newly diagnosed or relapsed/refractory AL amyloidosis (69). However, PIs administration is associated with an increased risk of systemic and pulmonary hypertension, HF, acute coronary syndrome, venous thromboembolism and cardiac arrhythmias (47, 70, 71). Interestingly, patients with MM receiving PIs plus IMiDs have an increased risk of thromboembolic events (both venous and arterial) compared with other therapies (hazard ratio [HR]: 1.31, confidence interval [CI]: 1.03–1.67) or PIs or IMiDs alone (HR: 1.37, 95% CI: 1.02–1.86) (72).

CTR-CT may be due to the induction of oxidative stress and endothelial dysfunction caused by the accumulation of proteinaceous species within CV tissues (73). Cardiomyocytes are susceptible to proteasome inhibition being non-proliferative cells. Therefore, proteolytic degradation represents a crucial strategy to reduce apoptosis associated with endoplasmic reticulum stress and to ensure long-term survival (74). Moreover, PIs may induce dysregulation of several intracellular pathways, leading to the activation of NFκB, impairment of endothelial nitric oxide synthase, mitochondrial dysfunction and fibrosis (47).

#### Bortezomib

Bortezomib is a first-generation PI which reversibly binds to the β5 proteasome subunit. Bortezomib has revolutionized the treatment of AL amyloidosis, and it is currently included in the CyBorD regimen as first-line therapy for patients non-eligible for ASCT (3). Carriers of the *t*(11;14) mutation show a worse response to bortezomib-based schemes (75). Hypertension is a common (1%–10%) AE associated with bortezomib, followed by uncommon (0.1%–1%) toxicities, such as hyperglycemia or diabetes mellitus, HF, atrial fibrillation and venous thromboembolism; while myocardial infarction and pulmonary hypertension are even rarer (<0.1%) (6). Atrial arrhythmias may be more frequent following intravenous injection compared to subcutaneous bortezomib (76).

In a phase I/II trial on patients (*n* = 31) with relapsed AL amyloidosis, those treated with bortezomib twice-weekly had an increased risk of cardiac dysfunction (23%), defined as >10% reduction in LVEF, compared to patients treated weekly (9, 77). Bortezomib has been associated with HF, complete heart block and ischemic heart disease, but studies on larger cohorts failed to demonstrate these associations (78–84). In a metanalysis of 25 clinical trials on various malignancies (MM, lymphoma, non-small-cell lung cancer, Waldenström's macroglobulinemia and ovarian cancer), treatment with bortezomib was not associated with a significant risk of CV toxicity (HR: 1.14, 95% CI: 0.82–1.62) compared to control medications, with a mortality rate related to CTR-CT of 3%. However, patients with MM had a greater incidence of cardiotoxicity (decline in LVEF, HF, cardiac arrest and arrhythmias) compared to other subgroups (4.3% vs. 2.3%). Interestingly, bortezomib monotherapy resulted in a higher frequency of CTR-CT compared to bortezomib combinations (85). A retrospective analysis of phase II and III trials involving MM patients treated with bortezomib confirmed that bortezomib does not significantly increase the risk of cardiotoxicity (86). Peripheral neuropathy is a common reason for bortezomib discontinuation and patients with lung disease should be monitored because of a small risk of pulmonary toxicity (87).

#### Carfilzomib

Carfilzomib is a second-generation PI which irreversibly binds to the β5 subunit, resulting in a more sustained inhibition of proteasome than bortezomib. Carfilzomib shows an increased efficacy and toxicity compared to other PI (3). Hypertension is a very common (≥10% incidence) AE associated with carfilzomib, while hyperglycemia or diabetes mellitus, HF, atrial fibrillation, venous thromboembolism and pulmonary hypertension are classified as common toxicities; myocardial infarction is uncommon (6).

CV and renal toxicity often contraindicate carfilzomib in AL amyloidosis. In a phase I/II study conducted on relapsed or refractory AL patients (*n* = 28) with non-severe disease (MAYO stage ≤2) receiving 8 cycles of carfilzomib during 16 months, the regimen was effective and feasible but cardiac and renal toxicities were common, requiring cautious monitoring (88).

Carfilzomib may induce CTR-CT in a significant proportion of patients. Among 526 patients with relapsed or refractory MM, those treated with carfilzomib had a significant risk of developing cardiac AEs (22%), such as HF (7.2%), arrhythmias (13.3%), and ischemic heart disease (3%) (89). In a real-life study conducted on patients (*n* = 22) treated with previous lines of MM therapy, 23% showed symptoms of HF (90). Another real-world analysis of carfilzomib-related CV adverse events in patients with MM (*n* = 7,330 subjects with 815 on carfilzomib) reported an increased risk of HF, ischemic heart disease and hypertension (all *p* < 0.001) than non-users. In the same study, pulmonary hypertension was diagnosed in approximately 1% of patients administered with carfilzomib (91).

The phase III ASPIRE trial, which compared the effects of carfilzomib with lenalidomide and dexamethasone to lenalidomide and dexamethasone alone in patients (*n* = 792) with relapsed MM, found that the carfilzomib group had better progression-free survival but enhanced frequency of CTR-CT (92). A meta-analysis of 24 studies involving patients with MM confirmed that carfilzomib is associated with a high risk of CV adverse events (18%) in a dose-dependent manner. In particular, HF (4.1%) and hypertension (12.2%) were the most frequent AEs, compared to arrhythmias (2.4%) and ischemic heart disease (1.8%). Dyspnea (23.9%) and peripheral edema (24.7%) were frequently detected (93). A recent systematic review including 45 studies on patients with MM treated with carfilzomib-based regimens reported that the incidence of HF, peripheral edema, hypertension and ischemic heart disease was 5.1%, 20.7%, 13.2% and 4.6%, respectively. In addition, no differences in terms of toxicity were detected when carfilzomib was used as a single agent vs. combination therapy (94). Carfilzomib may also cause significant corrected QT interval prolongation compared with other PIs, thus increasing the risk of ventricular arrhythmias and sudden cardiac death (95).

In the observational PROTECT study, patients with relapsed MM treated with carfilzomib and bortezomib (*n* = 95) were monitored for CTR-CT through cardiac biomarkers and echocardiography for 18 months. The reported AEs were similar to other studies, but the majority were transient, and treatment discontinuation was often not required (71). Finally, another real-life study comparing carfilzomib-dexamethasone vs. carfilzomib-lenalidomide-dexamethasone in MM patients (*n* = 109) reported that the former regimen was associated with an increased risk of CV events. However, the association was not significant after adjusting for confounding factors, suggesting that the enhanced CV toxicity in patients under the carfilzomib-dexamethasone scheme was related to their greater frailty (96). Finally, some reports highlight the association between carfilzomib and pulmonary hypertension in MM, probably due to the less vasodilator effect mediated by nitric oxide (97).

#### Ixazomib

Ixazomib is a second-generation PI with oral bioavailability and poor neurotoxic effects compared to bortezomib. Like bortezomib, ixazomib reversibly binds to the β5 proteasome subunit, with additional inhibition of β1 and β2 subunits at higher doses (3, 98). Ixazomib frequently results in peripheral edema (up to 18%) and hyperglycaemia when combined with lenalidomide or pomalidomide and dexamethasone (6).

In a phase I/II study including relapsed or refractory AL amyloidosis patients (*n* = 27), treatment with ixazomib was associated with a significant risk of NYHA class 3 dyspnea and fatigue (15%), but atrial fibrillation, congestive HF and pleural effusion were also common findings (7%), with 10% of patients manifesting NYHA class 3 HF (99). The phase III TOURMALINE-AL1 trial, comparing ixazomib plus dexamethasone to the treatment of choice (dexamethasone with/without melphalan, cyclophosphamide, lenalidomide or thalidomide) in patients with relapsed or refractory AL amyloidosis (*n* = 168), demonstrated that ixazomib-dexamethasone was well tolerated, with a similar safety profile between the two groups. Patients treated with ixazomib showed an increased risk of cardiac arrhythmias (26% vs. 15%), but the Authors assessed that the difference was mainly due to the greater frequency of non-drug-related atrial fibrillation (8% vs. 1%) (100). Although the study did not meet the primary endpoints of hematologic response, further analysis revealed that ixazomib administration is associated with similar or trended better quality of life and symptoms manifestation, compared with control regimens (101). In a phase I/II study, ixazomib-cyclophosphamide-dexamethasone was safe and well tolerated in patients with a new diagnosis of AL amyloidosis (102).

In the phase III TOURMALINE-MM1 trial, which evaluated ixazomib vs. placebo in combination with lenalidomide and dexamethasone in patients with relapsed or refractory MM (*n* = 722), CV adverse events (hypertension, HF, arrhythmias and ischemic heart disease) were not significantly different between the two arms. An important limitation of the study refers to the exclusion of patients with CV symptoms (hypertension, HF, unstable angina or ischemic heart disease) in the 6 months preceding the trial (103). A pharmacovigilance study comparing ixazomib with other approved drugs for MM reported an increased risk of atrial fibrillation (HR: 1.9, 95% CI: 1.5–2.3) among ixazomib users (104).

### Immunomodulatory drugs

IMiDs refer to a group of compounds that includes thalidomide and its derivatives (lenalidomide and pomalidomide). The anti-cancer mechanisms of IMiDs rely on direct cytotoxicity and indirect modulation of the neoplasm microenvironment, with effective antiangiogenic and immunostimulatory features (3). IMiDs carry a black box warning for fetal malformations, risk in pregnancy, hemotoxicity, arterial and venous thromboembolic events (48). For this reason, patients are usually recommended to thromboprophylaxis with aspirin or anticoagulants (105).

Treatment with IMiDs usually exhibits poor response in patients with AL amyloidosis, but represents an important element of combination therapy (25). Thalidomide and pomalidomide are also associated with sinus bradycardia (106). Finally, a network metanalysis demonstrated that the incidence of AEs is greater among patients treated with combined IMiDs and PIs, compared to IMiDs alone, despite the difference was not significant (HR: 1.47; 95% CI: 1.19–1.82) (107).

Moreover, therapy with lenalidomide and pomalidomide may result in transient NPs elevation and worsening of renal function, thus requiring cautious monitoring (108, 109). Patients treated with IMiDs drugs often show a discrepancy between haematologic and/or cardiac response and adverse NPs elevation, which is probably due to a chemotherapy-related CV effect or fluid retention (110). In a study including AL patients (*n* = 68) treated with lenalidomide and dexamethasone, 86% of subjects experienced a >30% increase in BNP levels after therapy (111). Another study (*n* = 106) reported that treatment with lenalidomide or pomalidomide was associated with higher risk of NT-proBNP increase compared with MelDex regimen (58% vs. 29%), but there was no association between FLC and NT-proBNP response (112).

IMiDs bind to cereblon, which is the substrate receptor of a component of the E3 ubiquitin ligase, that triggers proteasome-mediated degradation of the IKZF1 and IKZF3 transcription factors, resulting in increased production of interleukin-2 and other cytokines known to modulate T cell activity (113, 114). It has to be fully understood if these biochemical pathways are linked with CV toxicity, but IMiDs are generally believed to cause damage to endothelial cells, enhanced platelet aggregation, and higher von Willebrand factor levels (115).

#### Thalidomide

Thalidomide is a glutamic acid derivate largely used as an antiemetic for sickness during pregnancy until the 1960s, when its teratogenic potential was recognized, and the drug was withdrawn (3). The discovery of thalidomide's immunomodulatory and anti-angiogenetic effects led to its re-evaluation as an anti-cancer therapy (116). Thalidomide is associated with a high risk of neurological and gastrointestinal toxicity. Despite some promising results in terms of hematologic and organ response, the significant toxicities contraindicate its utilization in patients with AL amyloidosis (25, 117). Thalidomide therapy commonly results in HF and venous thromboembolism, while atrial fibrillation, myocardial infarction and arterial thromboembolism are uncommon findings (6).

#### Lenalidomide

Lenalidomide is generally effective, especially in combined therapies, but is poorly tolerated at the full 25 mg daily dose in patients with AL amyloidosis. Therefore, a reduced starting dose (5 mg) and closer monitoring are recommended (3, 6, 25). Notably, lenalidomide requires a dose adjustment based on renal status, since it is excreted through urine (3). Venous thromboembolism is a very common finding among patients treated with lenalidomide, as suggested by a trial evaluating lenalidomide plus high-dose dexamethasone vs. lenalidomide plus low dexamethasone in patients with a new diagnosis of MM (*n* = 445) (118). Hypertension, hyperglycemia, HF, atrial fibrillation and myocardial infarction are also common AEs (6). Arterial thromboembolism is uncommon. In a long-term follow-up of relapsed or refractory MM patients (*n* = 704) included in phase III trials, the risk of myocardial infarction and cerebrovascular events was higher in patients treated with lenalidomide-dexamethasone compared with placebo-dexamethasone (1.98% and 3.4% vs. 0.57% and 1.7%, respectively) (119).

The combination of PIs, lenalidomide, and steroids (lenalidomide-bortezomib-dexamethasone or ixazomib-lenalidomide-dexamethasone) often led to a hematological response, but the regimen was not well tolerated, leading to a high rate of therapy discontinuation despite low-dose lenalidomide (120, 121). Indeed, patients manifested a high risk of AEs, such as fluid overload (33.3%), arrhythmias (13.3%), and renal dysfunction (6.6%) (121). Lenalidomide is not significantly associated with peripheral neuropathy in AL amyloidosis patients, thus lenalidomide-based schemes may be considered for patients with neurologic involvement (25).

#### Pomalidomide

Pomalidomide is a third-generation IMiD which shows a safer renal profile and better tolerability compared to lenalidomide (3). Hypertension, atrial fibrillation, venous and arterial thromboembolism, and myocardial infarction are common findings (6). In a phase I/II trial including patients (*n* = 27) previously treated for AL amyloidosis, no thromboembolic events were registered, but kidney dysfunction and severe fatigue occurred in 26% and 18%, respectively (122). In a large retrospective series of relapsed or refractory AL amyloidosis patients (*n* = 153) treated with pomalidomide-dexamethasone, 33% of subjects showed severe AEs, including HF (7%), atrial fibrillation, suspected transient ischemic attack and atrial sinus block (with subsequent pacemaker implantation). Moreover, 19% of patients needed a dose reduction due to mild cytopenia (123).

### Monoclonal antibodies

#### Daratumumab

Daratumumab causes cell death through several mechanisms, including complement-dependent cytotoxicity, antibody-dependent cellular phagocytosis, and antibody-dependent cell-mediated cytotoxicity, as well as direct cellular apoptosis (124). Hypertension and atrial fibrillation are common AEs (23).

The phase III ANDROMEDA trial included newly diagnosed patients with AL amyloidosis (*n* = 388) comparing traditional treatment with CyBorD vs. CyBorD plus subcutaneous daratumumab followed by daratumumab monotherapy for up to 24 cycles. The trial demonstrated the beneficial effects of daratumumab-CyBorD in terms of hematological response or survival free from major organ deterioration. The most common CV adverse events in the daratumumab group included cardiac failure (9.3% vs. 7.4%), syncope (5.2% vs. 6.4%), peripheral edema (3.1% vs. 5.9%), and hypokalemia (1.6% vs. 5.3%) (125). A recent metanalysis found that the most common severe AEs in AL amyloidosis are lymphocytopenia, HF (4%–13%), infection complications, pneumonia, fatigue (0%–9%), atrial fibrillation (0%–18%), neutropenia, and diarrhea (126).

#### Isatuximab

Isatuximab is a chimeric IgG1κ mAb directed against CD38 targeting a different epitope compared to daratumumab, although both have proapoptotic effects with similar molecular mechanisms (21, 127). Isatuximab has a good hematological response rate and a good CV safety profile in relapsed disease (128). Isatuximab is being evaluated in a phase II trial (NCT03499808) in patients with relapsed or refractory AL and in a phase I trial (NCT04754945) in patients with high-risk AL amyloidosis. Hypertension is a very common AE in the context of MM therapy, whereas HF and atrial fibrillation are common findings (6). The main reasons for discontinuation were AEs in 26% of patients, including severe toxicities such as lymphopenia (9%), lung infection (6%), and an infusion-related reaction (3%) (129). Among patients with relapsed or refractory MM, the phase III IKEMA trial demonstrated that treatment with isatuximab plus carfilzomib-dexamethasone vs. carfilzomib-dexamethasone resulted in a similar rate of CV adverse events, including grade ≥3 hypertension (20%), HF (7%), and ischemic heart disease (5%) (130).

#### Elotuzumab

Elotuzumab is a humanized IgG1κ mAb targeting the signaling lymphocytic activation molecule family member F7 (SLAMF7) glycoprotein, normally expressed by plasma cells and amplified in patients with MM. Elotuzumab mainly acts through direct activation of NK cells and antibody-dependent cell-mediated cytotoxicity through the CD16 pathway (3). Hyperglicemia or diabetes are very common consequences of elotuzumab therapy; hypertension and venous thromboembolism are other common AEs (6).

A phase II trial (NCT03252600) is evaluating elotuzumab plus lenalidomide-dexamethasone with or without cyclophosphamide in relapsed AL amyloidosis. Small case series suggest that elotuzumab-lenalidomide-dexamethasone may prove safe and effective in previously treated AL patients (131). In the phase 3 ELOQUENT-2 trial, elotuzumab added to lenalidomide-dexamethasone in patients with relapsed or refractory MM was not associated with CV toxicities, and patients had a decreased risk of hypertension compared to those receiving lenalidomide-dexamethasone alone (1.3% vs. 2.2%) (132).

## Conclusions

Cardio-oncology represents a novel multidisciplinary field, which is the result of both advancements in cancer therapy and improvement in the prognosis of cancer survivors. Patients with AL amyloidosis require hematological treatment to eradicate the abnormal plasma cell clones through chemo-immunotherapy regimens usually administered in MM therapy. Despite the beneficial effects in terms of prognosis and organ response, cancer therapy may lead to CV toxicities and severe AEs, such as HF, arrhythmias and vascular disease. Optimal personalized care involving close collaboration between both cardiology and oncology specialists should be performed to prevent and limit complications. After a diagnosis of AL amyloidosis, patients should undergo a rigorous evaluation to determine the feasibility and risk of ASCT, which unfortunately can be performed in a small minority of subjects. Patients not eligible for transplant are preferentially treated with daratumumab-CyBorD, which represents the most effective and safe strategy. Regimens including other PIs, such as carfilzomib, and IMiDs, such as lenalidomide, may prove useful in terms of organ response, especially among patients where first-line options are not feasible, but CV adverse events need to be cautiously monitored.

Considering that CV toxicity may compromise therapy due to interruption of hematological treatment, a baseline risk stratification assessment is crucial before starting with therapy. Patients with AL amyloidosis and cardiac involvement are considered at very high CV risk because of the cardiac amyloid infiltration, thus requiring close cardiologic monitoring (every 3–6 months). On the other side, a periodical CV risk assessment after therapy should be performed in order to both monitor AL disease progression and prevent the onset of long-term therapy-related toxicities.

Over the last decades, the prognosis of patients with AL amyloidosis has improved significantly, highlighting the importance of cardiac protection as well as the prevention of CTR-CT. Finally, the understanding of the biochemical basis of CV toxicity may lead to the implementation of selective agents which counteract the toxic effects of traditional regimens. Future research is warranted also to elaborate and validate novel prevention and surveillance options capable of attenuating therapy-related CV toxicity in the setting of AL amyloidosis.
